# Effect of low-frequency acupuncture on muscle and fascia stiffness: examination with or without intervention

**DOI:** 10.3389/fresc.2024.1324000

**Published:** 2024-12-12

**Authors:** Toshihiro Maemichi, Masatomo Matsumoto, Shigeru Meguriya, Atsuya Furusho, Takashi Yamashita, Toshiharu Tsutsui, Tsukasa Kumai

**Affiliations:** ^1^ Faculty of Sport Sciences, Waseda University, Saitama, Japan; ^2^ Institute of Life Innovation Studies, Toyo University, Saitama, Japan; ^3^ Graduate School of Sport Sciences, Waseda University, Saitama, Japan; ^4^ Department of Medical Rehabilitation, Kuwana City Medicine Center, Mie, Japan; ^5^ Acupuncture and Physical Therapy Teacher Training School, Tsukuba University, Tokyo, Japan; ^6^ Tokyo Verdy, Inc., Tokyo, Japan; ^7^ Graduate School of Sport and Health Studies, Hosei University, Tokyo, Japan

**Keywords:** ankle dorsiflexion, fascia, low-frequency acupuncture, muscle, shear wave elastography, ultrasound, needling

## Abstract

**Background:**

Low-frequency acupuncture is used to maintain skeletal muscle flexibility and improve joint range of motion; however, its definite effects are unclear. This study aimed to determine the effects of low-frequency acupuncture on muscle and fascial stiffness and ankle dorsiflexion range of motion.

**Methods:**

The participants included 12 randomly selected healthy adults. The medial head of the gastrocnemius muscle was selected as the target muscle, and changes in hardness and dorsiflexion range of motion of the ankle joint in the deep fascia, muscle, and deep intermuscular fascia of the same region were measured before and after low-frequency acupuncture intervention. Acupuncture needles were inserted until they passed through the deep intermuscular fascia and electrically stimulated at a frequency of 2 Hz for 15 min. The 12 right legs were the intervention legs, and the 12 left legs were the non-intervention legs.

**Results:**

In the intervention leg, hardness increased in the deep fascia immediately after low-frequency acupuncture, but decreased in all regions (deep fascia, muscle, and deep intermuscular fascia) after 15 min. The rate of change in hardness was the greatest in the muscles and deep intermuscular fascia. Additionally, the ankle's dorsiflexion range of motion increased after 15 min. In contrast, the non-intervention leg showed no significant changes in stiffness or ankle dorsiflexion angle.

**Conclusions:**

Low-frequency acupuncture may decrease muscle stiffness and improve fascial gliding. The change in hardness tended to be greater in the deeper areas.

## 1 Introduction

Skeletal muscle stiffness and joint range of motion represent the body's flexibility. While flexibility is an important factor in preventing trauma and injury, excessive flexibility can compromise stability, leading to an increased risk of injury. Thus, achieving an optimal balance between flexibility and stability is essential for both performance and injury prevention. Athletes strive to maintain skeletal muscle stiffness and joint range of motion, which reportedly change owing to fatigue and other factors, in an appropriate state through various methods. Acupuncture is one of the methods used in sports medicine (1). The usefulness of acupuncture for sports injuries and disorders has been reported, particularly for the knee (2), hip (3, 4), and ankle (5, 6). It has also been proven effective in relieving skeletal muscle pain and fatigue, including the delayed onset of muscle soreness after sports activities (7–9). Acupuncture is not only often used for the treatment of injuries and disorders but also for conditioning purposes. In recent years, low-frequency acupuncture, in which needles are inserted into the body as electrodes, has gained considerable prominence (10, 11). Electrical stimulation is a physical therapy widely applied clinically in the rehabilitation field, and its main effects include induction of muscle contraction, pain control, promotion of healing in cases of difficult-to-heal wounds, muscle strengthening and re-education, reduction of muscle spasms, and transcutaneous penetration therapy (12–16). Along with these effects, low-frequency acupuncture has been used to improve localized areas of skeletal muscle stiffness and relieve tension. However, it is unclear whether it can indeed elicit changes in stiffness or range of motion. Acupuncture can also be treat deep tissues that are challenging to address manually, such as through massages, as the needles can stimulate these deeper layers. However, potential differences in the effects of acupuncture needles according to the depth of penetration into the muscle have not been assessed, and there have been no previous reports examining the effects of acupuncture needles and the extent of these effects according to the targeted tissue.

Conventionally, the hardness of skeletal muscle has been evaluated using palpation from the body surface, an indentation-type hardness measuring instrument, among other techniques. Additionally, the term “muscle hardness” often refers to the resistance force (17–19) provided by the muscle against vertical pressure, a concept that has been frequently documented. However, such evaluations can be notably unreliable as they are largely subjective, relying on the palpator's experience, and can be influenced by various tissues—from subcutaneous fat to fascia—up to skeletal muscle. While much of the literature discusses the effects of acupuncture and moxibustion in relation to these scenarios, there are no prior reports that thoroughly examine which tissues are affected by acupuncture and to what degree.

Shear wave elastography (SWE) is an objective and quantitative method used to evaluate the stiffness of biological tissues. SWE is used for imaging quantitative hardness distribution by measuring the propagation velocity of shear waves generated when microvibrations are applied to the tissue. Because of SWE's real-time nature, non-invasiveness, and relative simplicity, attempts to measure the mechanical properties of muscles, tendons, and fascia using this technique have already been conducted and were shown to provide highly reliable measurements (20–25). Herein, we aimed to evaluate the effects of low-frequency acupuncture on muscle and fascia stiffness and on the dorsiflexion range of motion of the ankle joint using SWE. We hypothesized that low-frequency acupuncture would decrease muscle and fascial stiffness and increase the range of motion.

## 2 Materials and methods

### 2.1 Participants

Twelve healthy young men (age 28.5 ± 4.2 years, height 168.3 cm ± 7.7 cm, weight 62.2 ± 11.1 kg) participated in this study (ACU). Additionally, the 12 left legs, which were not subject to low-frequency acupuncture, were designated as non-interventional legs (CON). The sample size was determined using GPower 3.1 software. Based on the study design, 12 participants were required to achieve a statistically significant result, assuming an effect size of 0.5, an alpha level of 0.05, and a statistical power of 80%.

The inclusion criteria were: (1) healthy males aged 20–35 years; (2) no history of musculoskeletal disorders; (3) no contraindications to acupuncture therapy. The exclusion criteria were: (1) performed strenuous physical activity or consumed alcohol 48 h before the measurement; (2) history of surgery or major trauma to the lower limb or foot; (3) current orthopedic injury to the lower limb or foot; (4) rheumatic diseases such as osteoarthritis, gout, or rheumatoid arthritis; (5) systemic diseases such as diabetes and/or connective tissue disorders. In this study, we focused on male participants to reduce confounding variables, taking into account hormonal fluctuations that could potentially influence muscle and fascia stiffness

This study was approved by our Institutional Human Research Ethics Committee for research involving Waseda University personnel and was conducted in accordance with the Declaration of Helsinki (Approval No. 2021-498). Written informed consent was obtained from all participants before data collection.

### 2.2 Protocol

Low-frequency acupuncture was applied to the medial head of the gastrocnemius muscle and rectus femoris muscle. Elasticity measurements of the deep fascia (DF), muscle, and deep intermuscular fascia (DIF) of the same area were performed before (PRE), immediately after (POST), and 15 min after (15 min) the application of low-frequency acupuncture using an ultrasound imaging device. Then, the maximum dorsiflexion range of motion of the ankle joint was measured before (PRE) and 15 min after the electric current was applied.

### 2.3 Ultrasound measurements

**Figure 1:**
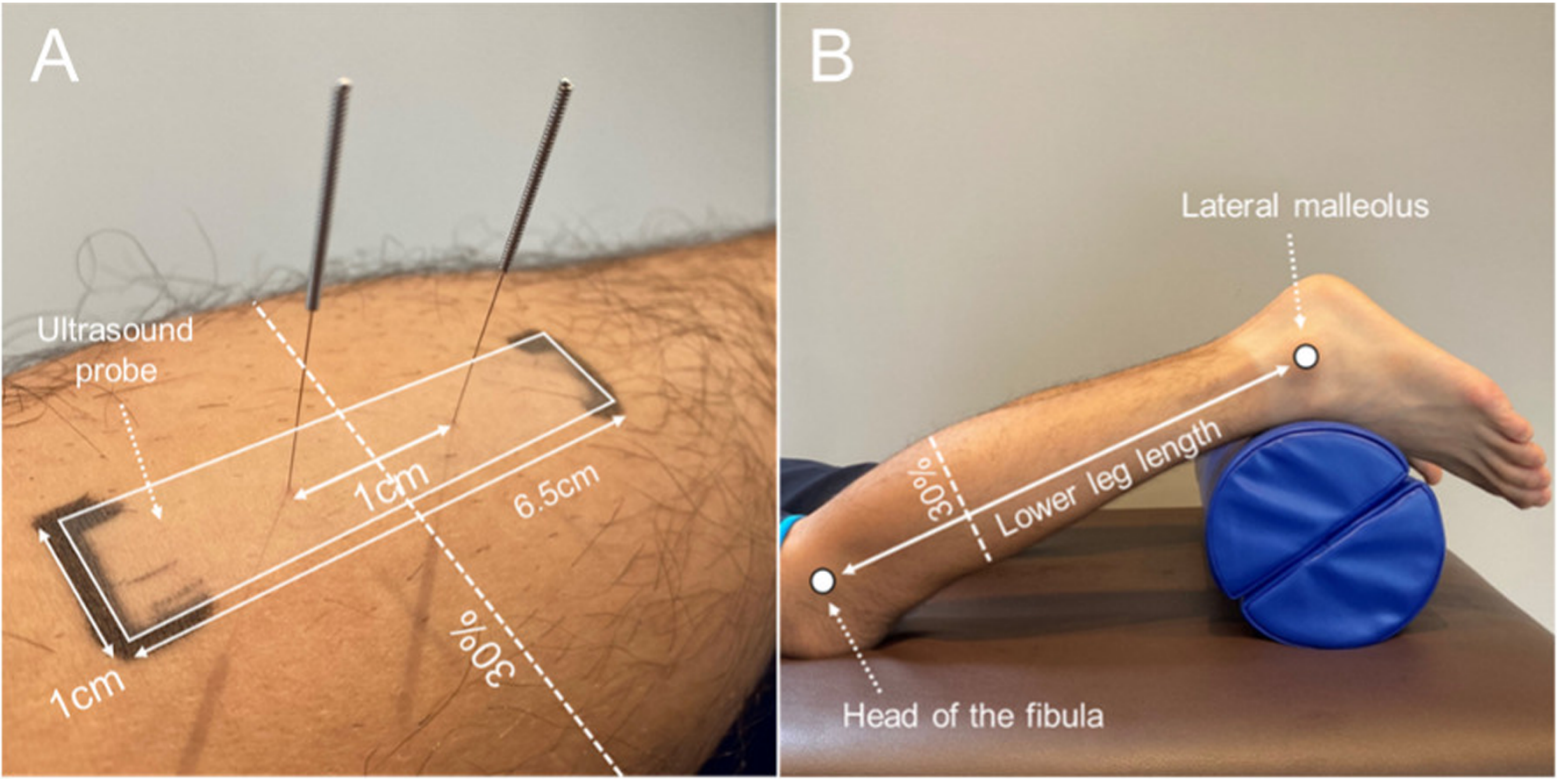
**(A)** A marker (6.5 cm × 1.0 cm) aligned with the ultrasound probe placed at the proximal 30% site of the lower leg length (lateral knee joint cleft to external capsule). **(B)** Lower extremity position during measurement and low-frequency electroacupuncture.

**Figure 2:**
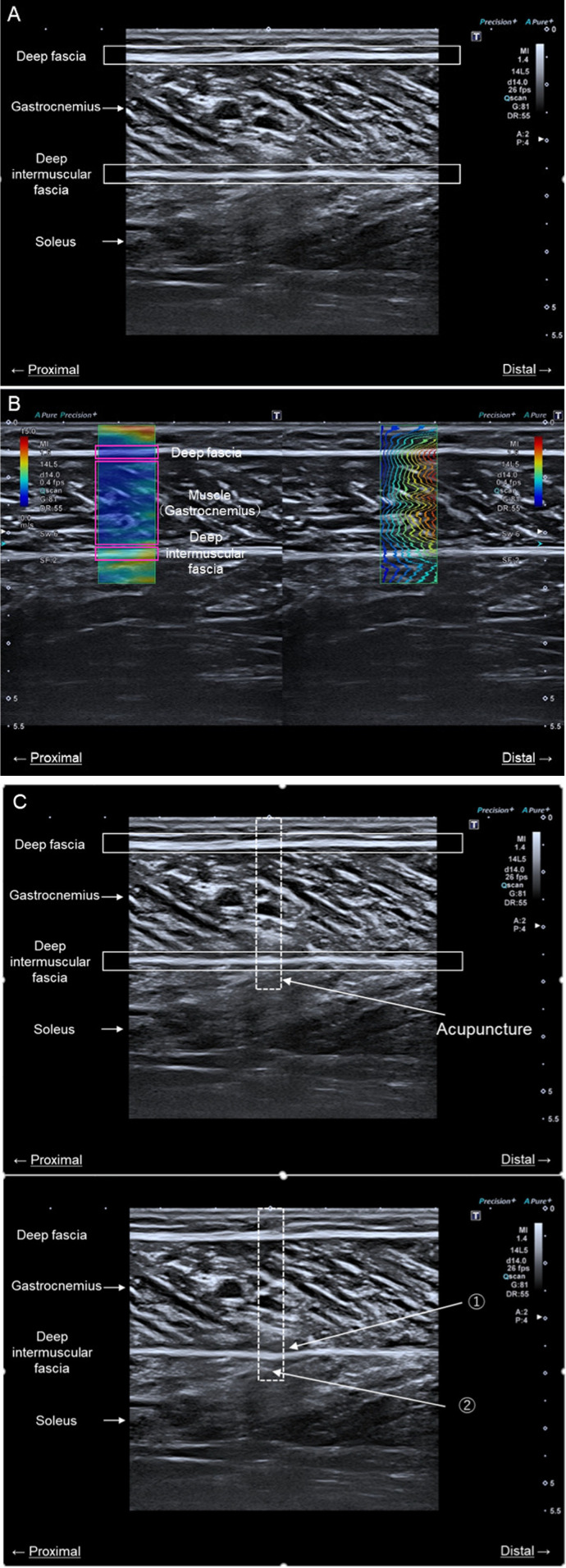
Ultrasound measurements. The image in **(A)** is an ultrasound image of the medial head of the gastrocnemius muscle. The image in **(B)** is an ultrasound image showing measurement of the shear wave velocity of muscle and fascia in the long axis. The image in **(C)** shows confirmation of the acupuncture needle insertion depth. The presence or absence of DIF passage was determined using an ultrasound imaging device to confirm the following two points: (1) the DIF flexed during acupuncture and (2) the acupuncture point was visible under the DIF. The dotted square line indicates the area where the acupuncture needle was inserted. DIF, deep intermuscular fascia.

A 5–14-MHz high-frequency linear probe was used in this study with an Aplio α ultrasound imaging system (Canon Medical Systems, Otawara, Japan). The medial head of the gastrocnemius muscle was imaged in the longitudinal direction with a marker (6.5 cm × 1.0 cm) aligned with the probe so that the center of the ultrasound probe was at 30% length of the proximal site of the lower leg (lateral knee joint cleft to the most proximal part of the external capsule) (Figure 1). The target limb was placed in a prone position, with a 15 cm-diameter pole placed under the ankle and knee joints in mild flexion and the ankle joint in mild plantar flexion (Figure 2).

SWE, a built-in function of ultrasound imaging systems, was used to measure the mechanical properties of the DF, muscle, and DIF. In principle, the ultrasound probe sends push pulses to displace the focused tissue deeply, and the restorative force of the tissue propagates laterally, generating shear waves. The probe measured the propagation speed of the shear wave by sending and receiving search pulses. SWV is a key indicator of tissue mechanical properties, with higher values generally indicating greater stiffness of the tissue. Conversely, lower SWV values suggest softer, more pliable tissue. In healthy muscle tissue, SWV values typically range from 1.0 to 4.0 m/s. Values outside this range may indicate pathological conditions or reduced muscle elasticity. This interpretation helps in understanding the significance of SWV measurements in our study. The shear modulus was calculated using the propagation velocity of the shear waves. The calculation formula is as follows:

(1) μ=ρ×ν2

(μ:Shearmodulus,ρ:Densityofmedium,ν:Speedofsound)

**Figure 3:**
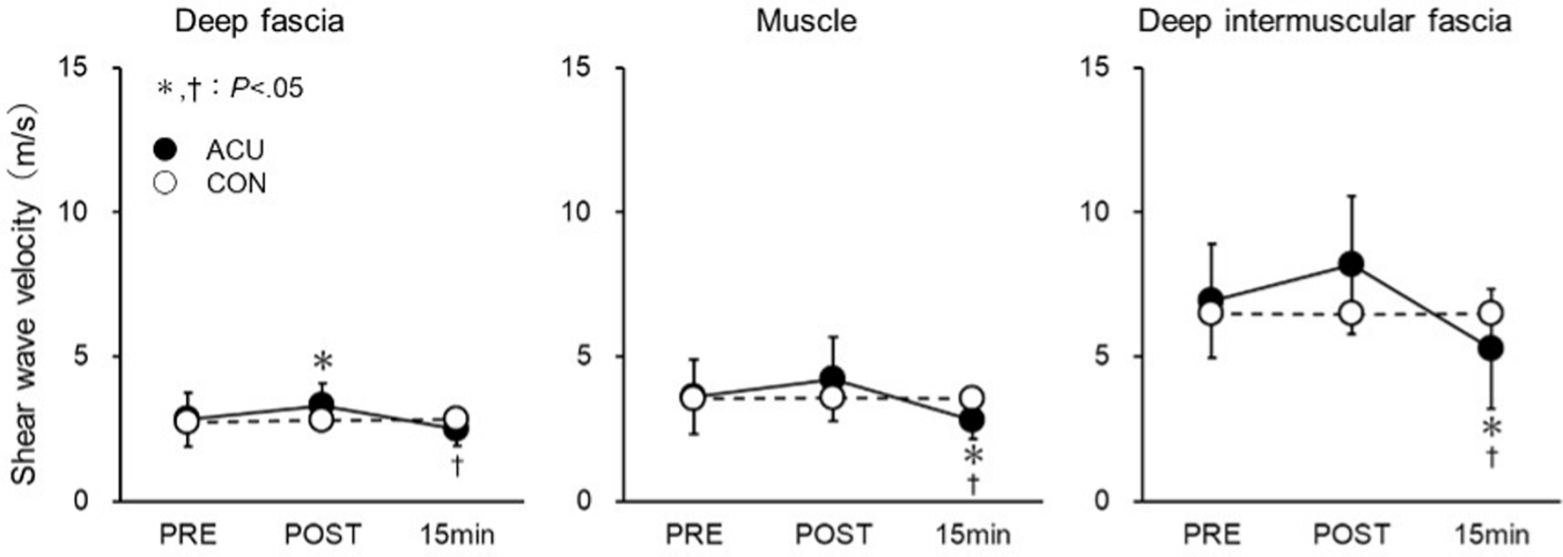
Shear wave velocity of the deep fascia (DF), muscle, and deep intermuscular fascia (DIF). Measurements were obtained before (PRE), immediately after (POST), and 15 min after the acupuncture plus low-frequency electrical stimulation. *Significantly different from PRE (*P* < 0.05). †Significantly different from POST (*P* < 0.05).

The shear wave propagation velocity was assumed to be constant at 1,000 kg/m^3^ in living tissue. The shear wave velocity (SWV) is a color elasticity map overlaid on the B-mode image, with hard tissues shown in red and soft tissues in blue. For quantitative elasticity measurements, the maximum, minimum, standard deviation, and average Young's modulus is displayed in kilopascals (kPa) or velocity units (m/s) by the software embedded in the system and were expressed as SWV values in this study. Because the ultrasound system used in this study was a commercialized system, the shape of the region of interest (ROI) was rectangular with a horizontal length of 1 cm, and all ROIs were standardized. Three images were taken at each site, and the DF, muscle, and DIF sites were surrounded by ROI from one image. The average of the values measured from the three images was used (Figure 3).

### 2.4 Low-frequency electric acupuncture

Disposable stainless-steel needles (50 mm long, 0.20 mm diameter) (M type SP, SEIRIN, Kyoto, Japan) were used. A low-frequency acupuncture energizer (Picorina, SEIRIN) at 2 Hz and an energization time of 15 min was used. The power output was adjusted according to the subject's tolerance range until the patient assigned an 8 on the numerical rating scale (0–10, where 0 = no pain at all and 10 = unbearable discomfort). The mean value of the output in this study was 3.1 ± 1.5 mA. The limb position during low-frequency acupuncture was the same as that during the ultrasound measurements (i.e., supine), with a 15 cm-diameter pole placed under the talofemoral joint to prevent movement of the ankle joint, the knee joint in slight flexion, and the ankle joint in slight plantar flexion. A video camera was used to monitor the procedure from the side to confirm that the joint angles did not change.

**Figure 4:**
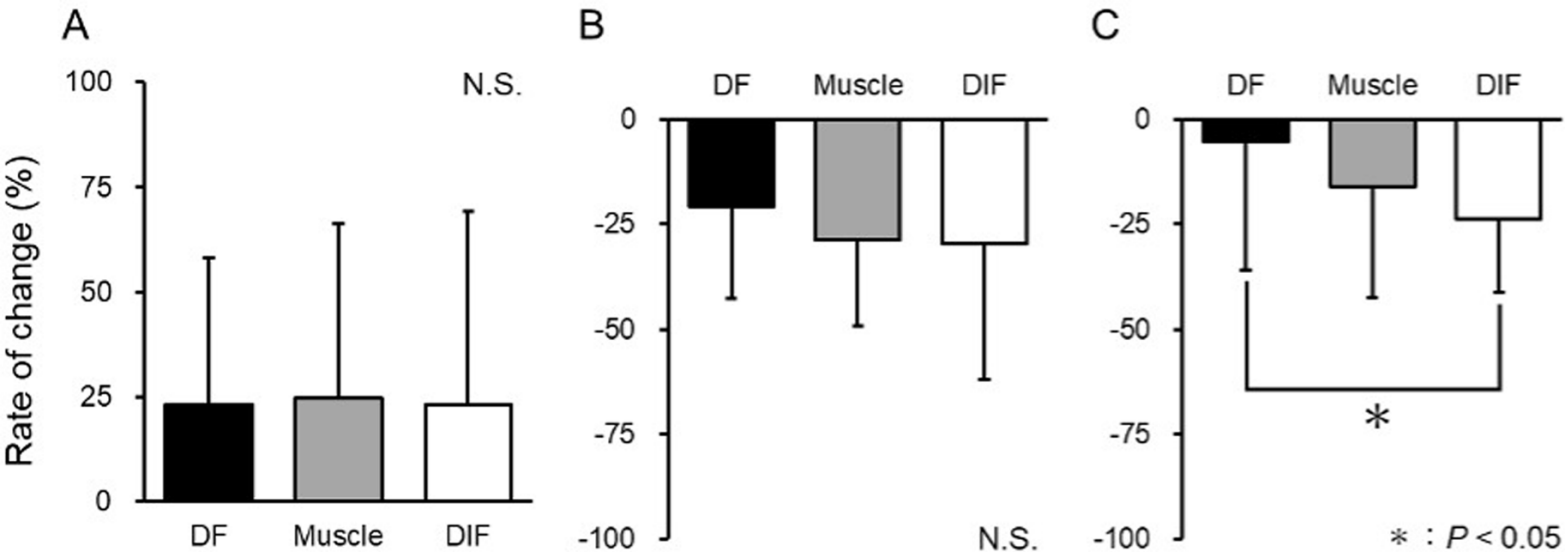
Rate of change in shear wave velocity after acupuncture plus low-frequency electrical stimulation. The rate of change was calculated at the deep fascia (DF), muscle, and deep intermuscular fascia (DIF). **(A)** Rate of change from PRE to POST. **(B)** Rate of change from POST to 15 min. **(C)** Rate of change from PRE to 15 min. *Significantly different from PRE (*P* < 0.05).

Acupuncture needles were inserted proximally and distally at 0.5-mm intervals within the 30% length of the proximal site of the lower leg (lateral knee joint crease to the external capsule), which was marked in accordance with the probe during ultrasound measurement. After sterilization of the same site with alcohol, two acupuncture needles were inserted 1 cm apart, perpendicular to the skin surface, and to a depth that ensured passage beyond the deep intermuscular fascia (DIF). This methodology allows for precise localization of the acupuncture points. The insertion intervals of 0.5 mm were utilized for fine adjustment, while the primary insertion of needles was conducted at 1 cm intervals to enhance the efficacy of the treatment. Additionally, the ultrasonic probe was confirmed in short-axis images and was inserted into the most bulging part of the muscle.The presence or absence of DIF passage was determined using an ultrasound imaging device to confirm the following two points: (1) the DIF flexed during acupuncture, and (2) the acupuncture tip was visible beneath the DIF (Figure 4).

### 2.5 Dorsiflexion range of ankle joint

The pull-sensor function of the manual muscle system (Mobie, SAKAImed, Tokyo, Japan) was used to measure ankle joint dorsiflexion range of motion. The patient was placed in a long sitting position with the knee joint in full extension and the lower back, thigh, and lower leg immobilized. A traction belt was applied from the big toe to the small toe, and traction was applied in the dorsiflexion direction from the mid-ankle joint position. Traction force was applied such that the patient experienced no discomfort in the lower leg and until they rated a score of 8 on the numerical rating scale (0: no pain at all, 10: unbearable discomfort). The angle of intersection between the fifth metatarsal line and the line connecting the head of the fibula to the external capsule was analyzed using ImageJ (National Institutes of Health, Bethesda, MD) as the ankle joint dorsiflexion range of motion. The traction force value in the PRE was recorded, and an equal traction force was applied during the post15 measurement. The mean traction force in this study was 16.2 ± 6.0 N/kg.

### 2.6 Statistical analyses

Statistical analysis was performed using statistical software SPSS software (SPSS Statistics 28, IBM Corp., Armonk, NY, USA). Intraclass correlation coefficient ICC (1.1) was determined for both intervention and non-intervention legs to evaluate the reliability of the SWV measurements. An evaluation of the coefficient of variation (CV) was also performed. CV was calculated by dividing the standard deviation (SD) of triplicate measurements by the mean. Descriptive data are shown as the mean ± SD. First, we clarified the changes in SWE over time for each tissue. A two-way analysis of variance was used to compare the PRE, POST, and 15-min SWV values of DF, muscle, and DIF between intervention and non-intervention legs. Where significant main effects and/or interactions were found, Dunn–Bonferroni or unpaired *t*-tests were performed as *post-hoc* tests, as appropriate. Next, we compared the rate of change in SWE between tissues. Shapiro–Wilk test was performed before comparing the rate of change in PRE, POST, and 15-min SWV values for DF, muscle, and DIF. The Kruskal–Wallis test was performed because normal distribution was not assumed.

The Dunn–Bonferroni method was performed as a *post hoc* test when the test result of the difference in measured values was significant. A two-way analysis of variance was used to compare PRE, 15-min ACU, and CON for the maximum ankle dorsiflexion range of motion between the intervention leg and the non-intervention leg. Where significant main effects and/or interactions were found, Dunn–Bonferroni or unpaired *t*-tests were performed as *post-hoc* tests, as appropriate. Effect sizes were also calculated using G*Power3.1. The significance level was less than 5%.

## 3 Results

The reliability results are listed in the Supplementary Table 1. The ICC values for DF, muscle, and DIF exceeded 0.95 across all practices.

Figure 3 shows the SWV values for each site. The Kruskal–Wallis test revealed significant differences in SWV values for DF, muscle, and DIF at all sites, but only in the intervention leg [(ACU) DF: *P* = 0.002; muscle: *P* = 0.001; DIF: *P* < 0.001].

No interaction was found between ACU and CON in DF. A *post hoc* Dunn–Bonferroni test was conducted for pairwise comparisons. In ACU, DF was significantly increased in POST than in PRE. However, a significant decrease was observed at 15 min compared with POST. No significant difference was noted between PRE and 15 min, with values being 2.82 m/s for PRE, 3.30 m/s for POST, and 2.51 m/s for 15 min (PRE-POST: *P* = 0.022; POST-15 min: *P* < 0.001; PRE-15 min: *P* = 0.246). In contrast, in CON there were no significant changes in DF when comparing POST to PRE or 15 min to POST.

In the muscles, no interaction was observed between ACU and CON. Using the Dunn–Bonferroni method as a *post-hoc* test for pairwise comparisons, there was no significant difference between PRE and POST in ACU. However, there was a significant decrease at 15 min when compared with both POST and to PRE. The respective values were: PRE: 3.62 m/s; POST: 4.22 m/s; 15 min: 2.79 m/s (PRE-POST: *P* = 0.185; POST-15 min: *P* < 0.001; PRE-15 min: *P* = 0.023). In CON, POST showed no significant change compared with PRE, and similarly, there was no significant change at 15 min compared with either POST or PRE.

For DIF, no interaction was found between ACU and CON. The *post hoc* Dunn–Bonferroni method revealed no significant difference between PRE and POST in ACU's DIF. However, a significant decrease was observed at 15 min compared both to POST and to PRE. The respective values were: PRE: 6.93 m/s; POST: 8.17 m/s; 15 min: 5.27 m/s (PRE-POST: *P* = 0.086; POST-15 min: *P* < 0.001; PRE-15 min: *P* = 0.016). In CON, there was no significant change in DIF from PRE to POST, and also no significant change at 15 min compared with either POST or PRE.

Figure 4 depicts the rate of change in the SWV value for ACU at each site. When evaluating the difference in SWV change rate for the intervals PRE-POST, POST-15 min, and PRE-15 min, a significant difference was found only for PRE-15 min (PRE-POST: *P* = 0.901; POST- 15 min: *P* = 0.253; PRE-15 min: *P* = 0.030). For the PRE-15 min interval where a significant difference was observed, the Dunn–Bonferroni method was applied for *post hoc* pairwise comparisons (*P* = 0.188; muscle-DIF: *P* = 1.000; DF-DIF: *P* = 0.031).

**Figure 5:**
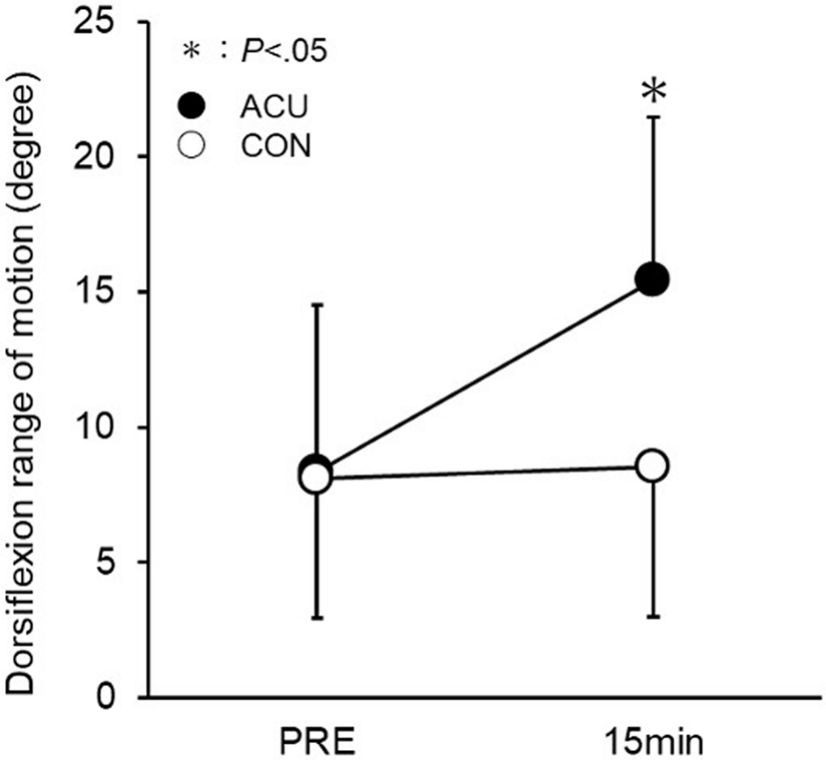
Changes in the maximum dorsiflexion range of motion of the ankle joint. The changes were measured from before (PRE) to 15 min after the acupuncture plus low-frequency electrical stimulation. *Significantly different from PRE (*P* < 0.05).

Figure 5 illustrates the changes in maximum ankle dorsiflexion range of motion. No interaction was found between ACU and CON. Employing the Dunn–Bonferroni method for *post-hoc* test pairwise comparisons, a significant increase in the maximum ankle dorsiflexion range of motion was observed from PRE to 15 min exclusively in ACU (PRE: 8.32 ± 6.19°, 15 min: 15.42 ± 6.06°, *d* = 1.14).

## 4 Discussion

This study aimed to assess the effects of low-frequency acupuncture on muscle and fascia hardness using SWE, which can objectively and quantitatively evaluate the hardness of biological tissues. While traditional methods, such as palpation or push-in hardness meters, have been used to assess muscle hardness, these methods have limitations due to their subjective nature and influence from surrounding tissues like subcutaneous fat and fascia (20–25). SWE provides an objective alternative; however, no direct cross-comparisons with previous evaluation methods were made in this study.

Most of the literature reporting the effects of acupuncture treatment is affected by the aforementioned problems, and no previous reports have examined the effects of acupuncture and the extent of these effects according to the tissue being targeted.

Immediately after low-frequency acupuncture, there was a significant increase in hardness in the deep fascia (DF) and a non-significant but increasing trend in hardness in other regions. Continuous electrical stimulation of the motor nerve-dominant muscles with muscle contraction is believed to cause muscle fatigue. Muscle stiffness increased in fatigued muscles as well as after exercise, suggesting that muscle stiffness may increase shortly after such activities. Additionally, because muscle fatigue temporarily maintains muscle tension, it is suggested that the muscle has been in a state of contraction immediately after energization, which may have resulted in an increase in hardness. However, 15 min later, the hardness decreased relative to both the pre-acupuncture and immediate post-acupuncture periods. Low-frequency acupuncture stimulation has been reported to increase muscle blood flow (26), and the improvement in muscle blood flow leads to the relaxation of muscle tension, which may have led to a decrease in hardness in this study (27, 28)^.^ It was also suggested that the change might not occur immediately but after a few minutes, as shown in the results of this study.

The rate of change in hardness was more pronounced in the deeper muscle and deep interfacial (DIF) than in shallower DF areas. Deep insertion of acupuncture needles increases local deep blood flow (29, 30), and applying an electric current further increases blood flow due to muscle pumping and other effects. The study indicates a significant decrease in muscle and DIF stiffness for similar reasons. In addition, a significant decrease in hardness was observed in the DIF between DF and DIF, which corresponded to the myofascial area. This suggests that low-frequency acupuncture may have had a greater effect at the acupuncture tip site and at deeper sites.

The ankle dorsiflexion range improved 15 min after low-frequency acupuncture. One possible factor is the decrease in muscle hardness associated with the increased blood flow in the muscles mentioned above. In this study, the medial head of the gastrocnemius, a pinnate muscle, was used as the target muscle for verification. We visually observed lateral movement in the DF (between the fat and muscle layers) and DIF (between the gastrocnemius and soleus muscles). In other words, we observed a misalignment (i.e., sliding) between the tissues in the DF and DIF areas during muscle contraction. Since it has already been shown that the distance traveled by the fascia increases as the stiffness of the fascial region decreases (31), it is possible that the stiffness of the fascial region in this study also decreased due to the improvement in sliding. In other words, the improvement in the dorsiflexion range of motion of the ankle joint in this study was not only due to a decrease in muscle stiffness but also due to an improvement in the sliding of the DF and DIF, suggesting that there is a significant contribution from the fascia as well.

It is challenging to derive concrete conclusions from this study's results, and it was difficult to clarify the mechanism since no similar studies exist for reference. However, the change in hardness at DIF was equal to or greater than that at the muscle site. It is clear from previous studies that mechanical stimulation of fascia, such as using hyaluronic acid, causes a decrease in hardness (32). Furthermore, it is undeniable that acupuncture and electricity may have caused some changes in the fascia in this study. Therefore, further studies are warranted to provide relevant information.

The results of this study revealed that the major changes in tissue hardness affected both muscles and fascia. The greatest strength of acupuncture is that the depth of insertion can be selectively changed. It can be expected that the hardness of the site can be improved by inserting the needle according to the deep adhesion or induration site that occurs after fracture or surgery and applying electricity. In addition, since not only the muscle but also the fascia part changed significantly, we believe that the method of selecting according to the purpose of the tissue is effective.

To clarify the mechanism of low-frequency acupuncture, we plan to increase the number of participants and consider implementing it for cases. In addition, we conducted a comparative study of non-invasive low-frequency acupuncture, such as surface electrification, and invasive low-frequency acupuncture, such as electroacupuncture. We clarified the advantages of using it despite the risks associated with invasiveness.

The limitations of our study are as follows: The sample size was small, and all participants were healthy. Therefore, the effects observed might differ when treating muscle fatigue. Future studies should target fatigued muscles. Although it has been reported that effects can vary depending on the stimulus intensity and frequency of the current (33, 34)^,^ our study only examined 2 Hz for 15 min, which is frequently used in clinical practice. Adjustments to the stimulus parameters should be explored in the future. Additionally, we did not discern the effects based on invasive vs. non-invasive methods, a comparison we intend to make in subsequent studies.

In conclusion, these findings carry clinical implications. First, low-frequency acupuncture contributes to changes in both muscle and myofascial elasticity. The results suggest that reducing muscle and myofascial elasticity can enhance the range of motion and prevent disability. Second, the change in elasticity was most pronounced at the acupuncture needle's tip. This suggests that acupuncture may be very effective in improving the elasticity of deep tissues that cannot be reached by manual techniques. In addition, it has been shown that adhesion occurs between tissues at the injury site after surgery or fracture. Acupuncture needling at the same site may effectively improve sliding between adhered tissues. This could potentially expedite an athlete's return to competition. Furthermore, by bringing the acupuncture needle tip site to the target tissue where elasticity improvement is desired, it may be possible to improve tissue elasticity selectively, not only in deep areas. Moving forward, we intend to conduct thorough validation for postoperative cases and situations aiming to enhance the range of motion.

## Data Availability

The original contributions presented in the study are included in the article/Supplementary Material, further inquiries can be directed to the corresponding author.
