# Supplementary material for: Effect of low-frequency acupuncture on muscle and fascia stiffness: examination with or without intervention
Source: Front Rehabil Sci. 2024 Dec 12;5:1324000. doi: 10.3389/fresc.2024.1324000 (PMC11670481; doi:10.3389/fresc.2024.1324000)
Supplement: Supplementary file 1 [file Table1.docx]

Supplementary Material

**Supplementary Table 1.** Reliability values of the measurements

|  |  | DF | | |  | Muscle | | |  | DIF | | |
| --- | --- | --- | --- | --- | --- | --- | --- | --- | --- | --- | --- | --- |
|  |  | PRE | POST | 15 min |  | PRE | POST | 15 min |  | PRE | POST | 15 min |
| ACU | ICC (1.1) | 0.97 | 0.95 | 0.99 |  | 0.99 | 0.99 | 0.96 |  | 0.98 | 0.99 | 1.00 |
|  | CV (%) | 0.33 | 0.24 | 0.23 |  | 0.36 | 0.34 | 0.23 |  | 0.28 | 0.29 | 0.39 |
| CON | ICC (1.1) | 0.99 | 0.98 | 0.99 |  | 0.99 | 0.99 | 0.99 |  | 0.99 | 1.00 | 1.00 |
|  | CV (%) | 0.36 | 0.30 | 0.31 |  | 0.36 | 0.34 | 0.26 |  | 0.30 | 0.30 | 0.35 |

The intraclass correlation coefficient (ICC) (1.1) was calculated using three intraday shear wave velocity measurements. The coefficient of variation (CV) was calculated by dividing the standard deviation of three repeated measurements by the average of the three measurement values.

DF, deep fascia; DIF, deep intermuscular fascia
